# Ex vivo rat heart normothermic perfusion with intermittent low flow and triiodothyronine

**DOI:** 10.3389/fcvm.2025.1607594

**Published:** 2025-10-03

**Authors:** Iordanis Mourouzis, Dimitris Anagnostopoulos, Vassiliki Brozou, Dimitris Kounatidis, Dimitris Giannoulis, Athanasios Lourbopoulos, Theodosios Saranteas, Constantinos Pantos

**Affiliations:** ^1^ Department of Pharmacology, University of Athens, Athens, Greece; ^2^ Department of Anaesthesia and Cardiovascular Critical Care, School of Medicine, Attikon Hospital, University of Athens, Athens, Greece

**Keywords:** crystalloid solution, preconditioning, normothermic perfusion, triiodothyronine, transplantation, heart

## Abstract

**Background:**

Ischemia-reperfusion injury remains the main constraint of normothermic perfusion. The present study investigated the potential of therapies targeting reperfusion injury such as triiodothyronine and interventions mimicking ischemic preconditioning (PC) to optimize normothermic crystalloid perfusion.

**Methods:**

Rat hearts were perfused for 6 h with constant flow, in a Langendorff mode and Krebs-Henseleit as perfusate with glucose being the only metabolic substrate, (NP, *n* = 9). PC treated hearts were subjected to 5 cycles of 40 min low flow perfusion followed by 20 min normal flow perfusion with either vehicle (PC, *n* = 11) or 60nM T3 (PC + T3, *n* = 10). Left ventricular end diastolic pressure (LVEDP), left ventricular developed pressure (LVDP), perfusion pressure (PP), and percentage of change of these parameters from baseline values were measured. The ratio of LV weight to body weight was calculated as index of tissue edema.

**Results:**

Baseline parameters were similar between groups. At the end of perfusion, no difference in LVDP was observed, LVEDP was significantly lower in PC and PC + T3 vs. NP, *p* < 0.05. PP was significantly lower in both PC and PC + T3 vs. NP hearts. *p* < 0.05. Both PC and PC + T3 significantly reduced tissue edema.

**Conclusion:**

Intermittent low flow mimicking ischemic preconditioning (PC) appears to optimize crystalloid based normothermic rat heart perfusion by limiting tissue edema and diastolic and vascular dysfunction.

## Introduction

Normothermic ex vivo heart perfusion is an emerging approach for organ preservation as an alternative to cold cardioplegia. Unlike cold ischemic preservation, the donor heart is kept in a beating, nearly physiologic state, and allows graft assessment before implantation (1). A growing body of evidence has established the safety and effectiveness of this technique (1–4). However, there are constraints that limit its wide use in clinical practice. The optimal composition of perfusate solution, the duration of perfusion and the potential use of normothermic perfusion after cold ischemia transportation remain yet undefined (5). Currently, perfusate composition is mainly based on the principle of optimizing the balance between oxygen demand and supply and blood based perfusates are used to enhance oxygen delivery in a metabolically active donor heart. Although this technique is proven to be effective, blood based solutions have different risks and unwanted effects and implicate a more complex application of the machine normothermic perfusion (6). Thus, optimization of ex vivo heart normothermic perfusion still remains a great therapeutic challenge.

Ischaemia-reperfusion injury is one of the main constraints of normothermic perfusion. Despite the restoration of flow and tissue oxygen availability, reperfusion injury occurs and cardiac function declines after long perfusion (7). This response is due to a number of changes that occur in cell homeostasis. Glucose metabolism is limited due to the uncoupling of glycolysis to oxidative phosphorylation (7–10). In addition, red blood cell deformability is impaired, cell aggregation is increased and may result in microvascular occlusion (11–13). Furthermore, endothelium and cardiac cells are damaged due to oxidative stress induced activation of pro-apoptotic pathways, such as the p38 MAPK intracellular signalling pathway (13–16). On the basis of this evidence, targeting reperfusion injury may be an attractive therapeutic modality for optimization of normothermic perfusion.

Historically, hormones and particularly thyroid hormone were shown to protect and preserve the donor heart (10, 17). Thyroid hormone appears to have differential actions on injured and non-injured myocardium. Furthermore, thyroid hormone sensitivity changes after myocardial infarction (18) and higher doses are required for thyroid hormone to be effective in patients with myocardial infarction (19, 20). In reperfused heart, triiodothyronine enhances coupling of glycolysis to oxidative phosphorylation (9, 10) and supresses the stress induced activation of p38 MAPK (21, 22). In addition, triiodothyronine prevents stress induced erythrocyte aggregation (23). Triiodothyronine has been shown to reduce reperfusion damage and improve cardiac function in experimental models of ischemia-reperfusion (21, 22) and in patients with acute myocardial infarction (19, 20). In line with this evidence, high dose continuous triiodothyronine administration optimized normothermic crystalloid based perfusion in an isolated rat heart model of perfusion (24). Notably, triiodothyronine and ischemic preconditioning share common cardioprotective mechanisms (22). It is therefore likely that interventions mimicking ischemic preconditioning could also improve normothermic perfusion. This could be of clinical relevance. Non pharmacological cardioprotective interventions may be a simple and feasible method to optimize normothermic perfusion. It is of note that controlled rewarming and normothermic perfusion with cell free solution is shown to be an effective method for human kidney preservation prior to transplantation (25).

Based on this evidence, the present study investigated whether intermittent low flow mimicking ischemic preconditioning (PC) alone or in combination with triiodothyronine could protect isolated rat hearts subjected to normothermic crystalloid based perfusion in a Langendorff mode.

## Materials and methods

### Animals

Wistar male rats, 16–24 weeks old, were used for this study. The rats were handed in accordance with the Guide for the Care and Use of Laboratory Animals published by the US National Institutes of Health (NIH Pub. No. 83­23, Revised 1996). The protocol of the study was approved by the Animal Care and Use Committee of Department of Pharmacology, Medical School, National and Kapodistrian University of Athens (license 120946/18-04-2019, *Ε*L 25BIOexp 10).

### Experimental protocol

A protocol of intermittent 5 cycles of 40 min low-flow perfusion followed by 20 min normal flow perfusion was applied as an intervention mimicking ischemic preconditioning. This intervention is designated as PC in the present study.

In order to investigate the potential effects of PC with and without the addition of T3 on ex vivo rat heart normothermic perfusion, the following experiments were performed

a. Hearts excised and subjected to an initial period of 30 min normothermic perfusion (stabilization period) in Langendorff apparatus with Krebs-Henseleit (KH) followed by 330 min of normal flow perfusion with KH buffer supplemented with vehicle (group NP, *n* = 9). Coronary flow was kept constant throughout the experiment.
b. Hearts excised and subjected to an initial period of 30 min normothermic perfusion (stabilization period) in Langendorff apparatus with KH buffer followed by another 30 min period of normal flow perfusion and PC protocol, (PC, *n* = 11). KH buffer was supplemented with vehicle at the end of stabilization period.
c. Hearts excised and subjected to an initial period of 30 min normothermic perfusion (stabilization period) in Langendorff apparatus with KH buffer followed by another 30 min period of normal flow perfusion and PC protocol, KH buffer was supplemented with 60nM T3 at the end of stabilization period (PC + T3, *n* = 10).

During stabilization all groups were treated the same.

### Anesthesia and recovery after normothermic perfusion

Rats were anaesthetized with ketamine HCl and heparin 1,000 IU was given intravenously. The hearts were rapidly excised, placed in ice-cold Krebs­-Henseleit (KH) buffer and mounted on the aortic cannula in Langendorff perfusion system within 60 s. Retrograde perfusion is already used in the existing perfusion machines approved for human heart perfusion (2, 5). Perfusion in the isolated rat heart system was achieved with oxygenated (95% O_2_/5% CO_2_) KH buffer as previously described (21, 24). The composition of KH buffer (in mmol/L) was the following: sodium chloride 118, potassium chloride 4.7, potassium phosphate monobasic 1.2, magnesium sulphate 1.2, calcium chloride 1.4, sodium bicarbonate 25, and glucose 11.

The pressure signal of the left ventricle (LV) was monitored in real time via a water filled balloon which was inserted in the LV cavity via the left atrium and connected to a pressure transducer. Left ventricular balloon volume was adjusted to produce an average initial left ventricular end-diastolic pressure of 6–8 mmHg in all groups and was held constant thereafter throughout the experiment. Thus, measurement of LV pressure was performed under isovolumic conditions. Data analysis software (IOX, Emka Technologies) was used in order to analyze and record the LV pressure and the perfusion pressure signals (24).

The perfusion apparatus was heated to ensure a temperature of 37 ^o^C throughout the course of the experiment. Hearts were paced at 320 beats/min with a Harvard pacemaker. Pacing was stopped during periods of low flow and re-established 5 min after coronary flow restoration.

At the end of perfusion period, the right ventricle was cut from the heart and the left ventricle (LV) was weighed. The ratio of LV weight to body weight was calculated as an index of tissue edema (26).

### Administration of triiodothyronine

3,5,3'-triiodothyronine (T3) was purchased from Sigma Chemicals (St Louis MO, USA). T3 was dissolved in ethanol with the addition of NaOH which results in T3 sodium salt. Then T3 was diluted in 0.9% sodium chloride buffer to create a stock solution with a concentration of 1 mg/ml and was kept at −20 °C. Before each experiment 40μl of this stock solution was added per liter of KH buffer to reach a final concentrationof 60nM T3. This dose is several times higher compared to doses used in clinical practice to treat hypothyroidism and restore T3 levels in blood to 1–2nM. The dose of 60nM T3 has previously been shown to be cardioprotective against hypoxic injury in an isolated rat heart experimental model and during normothermic perfusion (21, 24). T3 administration or vehicle (T3 diluent) was added to the perfusate after the first 30 min of perfusion (stabilization period) in the isolated heart apparatus.

### Measurement of mechanical function

Left ventricular function was assessed by recording the left ventricular developed pressure (LVDP, mmHg) and the positive and negative first derivative of LVDP (+dp/dt and –dp/dt). Diastolic function was assessed by monitoring isovolumic left ventricular end-diastolic pressure (LVEDP) as a measure of diastolic chamber distensibility. Perfusion pressure under constant flow conditions was used to assess coronary vessel resistance (PP, mmHg). Parameters were also expressed as percentage of change from baseline values.

### Statistics

Values are presented as mean (standard deviation). Normal distribution of variables was estimated with Shapiro–Wilk test of normality. Normally distributed data between groups were compared using one-way ANOVA. Paired samples *t*-test was used to compare measurements between stabilization and end of perfusion within the same group. Serial measurements of LVEDP and PP were also compared by mixed, repeated measures analysis of variance (mixed ANOVA) to test for the effect of treatment, time and the interaction (tests for “within-subjects” factor and “between-subjects” factor); the respective non-linear fit-curves were produced with non-linear fit analysis. When significant, differences within and between each group were tested by a *post hoc* analysis using Bonferroni correction for multiple comparisons. A two-tailed test with a *p* value less than 0.05 was considered significant. Analysis was performed using SPSS 23.0 and GraphPad 8 software.

## Results

### Cardiac function

**Figure 1:**
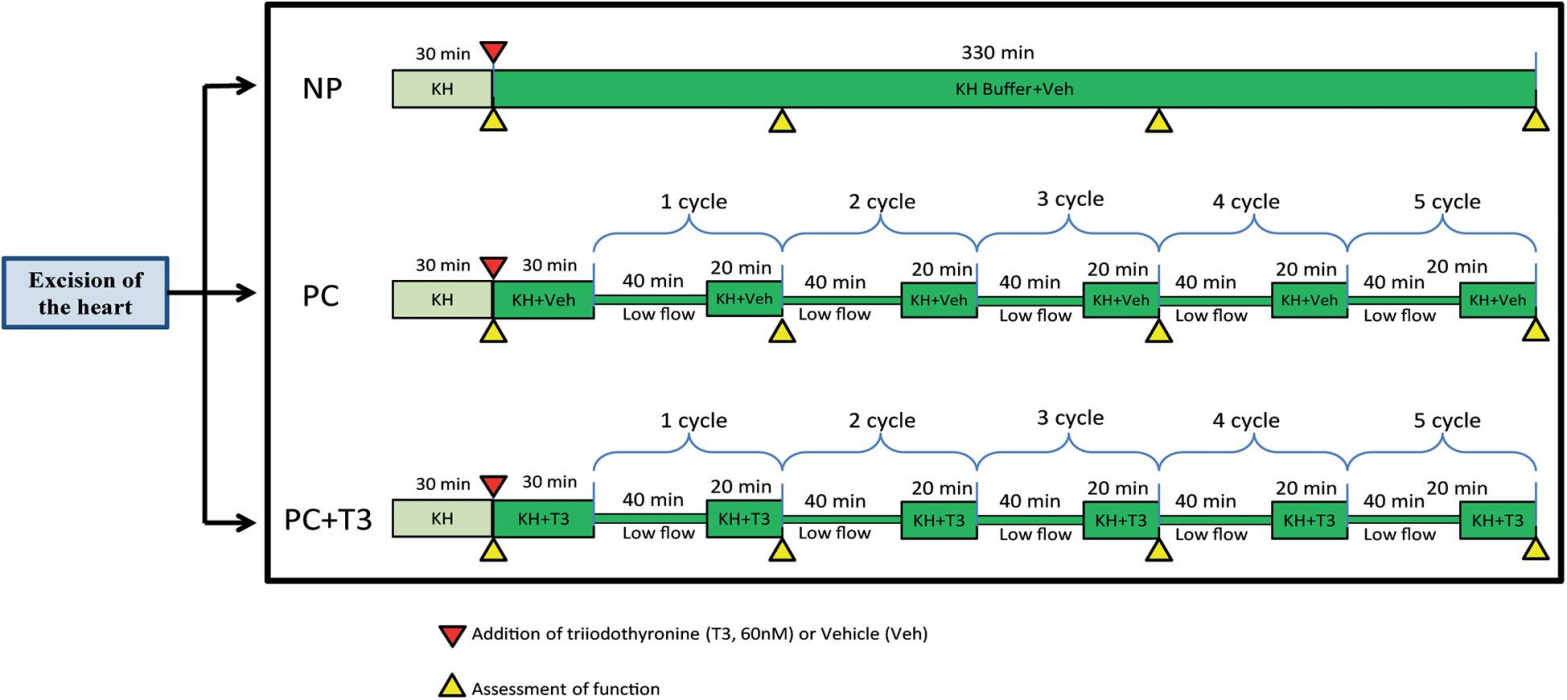
Schematic diagram showing the experimental design of the study.

In this study, rat hearts were perfused for 6 h with constant flow, in a Langendorff mode and Krebs-Henseleit as perfusate with glucose being the only metabolic substrate (NP), while PC treated hearts were subjected to 5 cycles of 40 min low flow perfusion followed by 20 min normal flow perfusion with either vehicle (PC) or 60nM T3 (PC + T3). Figure 1. The selection of the PC protocol and the perfusion duration was based on pilot studies (data not shown). The rationale was to establish a long duration protocol of perfusion with periods of low flow ischemia and reperfusion which is protective and does not result in irreversible damage. Flow was adjusted during the first 30 min of stabilization in order to achieve a mean perfusion pressure of 65–70 mmHg (normal flow) and was kept constant thereafter in group NP. In groups PC and PC + T3, flow was kept constant during the normal flow periods after the initial adjustment period. During periods of low flow perfusion, the rate of the peristaltic pump was decreased to 20% of normal flow and perfusion pressure was reduced to 20–24 mmHg. In accordance with the above, mean normal flow (L/min/g body weight) was 33 (9.8) for NP, 32 (4.5) for PC and 30 (3) for PC + T3 groups. During low flow periods, mean flow was 6.4 (0.9) for PC and 6.0 (0.6) for PC + T3 groups.

Left ventricular developed pressure (LVDP) and the rate of increase and decrease of LVDP (+dp/dt and -dp/dt) at the end of stabilization (baseline parameters) or the end of normothermic perfusion were not statistically different between groups Table 1. In NP group, LVDP decreased by 32.3% (11) % from baseline vs. 31.2% (8)% in PC group, *p* = 0.95. In PC + T3 group, LVDP decreased by 37.7% (11)% from baseline, *p* = 0.74 vs. NP.

**Table 1:** Left ventricular developed pressure (LVDP) and the rate of increase and decrease of LVDP(+dp/dt and -dp/dt) at the end of stabilization (baseline parameters)and the end of normothermic perfusion are presented. Data are presented as Mean (SD).

Groups	LVDP (mmHg)		+dp/dt(mmHg/sec)		-dp/dt(mmHg/sec)	
	30 min (Baseline)	End of perfusion (330 min)	30 min (Baseline)	End of perfusion (330 min)	30 min (Baseline)	End of perfusion (330 min)
NP (*n* = 9)	115.6 (9.2)	77.9 (12.0)	3,867 (688)	2,992 (290)	2,097 (201)	1,333 (191)
PC (*n* = 11)	113.2 (9.1)	77.9 (11.0)	3,614 (676)	3,010 (517)	1,961 (223)	1,310 (184)
PC + T3 (*n* = 10)	120.8 (8.7)	75.0 (12.9)	4,149 (440)	2,800 (391)	1,951 (206)	1,200 (166)

LVEDP baseline parameters were similar in all groups Table 2. At the end of perfusion, LVEDP was significantly increased in both in NP and PC group as compared to baseline, *p* < 0.05. Interestingly, in PC + T3 group, LVEDP at the end of perfusion was similar to baseline. The magnitude of LVEDP change from baseline (LVEDP %) was significantly less in PC and PC + T3 compared to NP group. In NP group, LVEDP increased 188 (94) % from baseline vs. 73 (98)% in PC group, *p* = 0.021 Table 2. In PC + T3 group, LVEDP increased only 8 (58)% from baseline, *p* = 0.0003 vs. NP Table 2.

**Table 2:** Left ventricular end-diastolic pressure (LVEDP) and perfusion pressure (PP) at the end of stabilization (baseline parameters) and the end of normothermic perfusion are presented. Data are presented as Mean (SD).

Groups	LVEDP (mmHg)			PP (mmHg)		
	End of Stabilization	End of perfusion	% Change	End of Stabilization	End of perfusion	% Change
NP (*n* = 9)	7.6 (0.3)	21.8 (7.0)*	188 (94)%	69 (5)	153 (47)*	120 (63)%
PC (*n* = 11)	7.7 (0.4)	13.4 (7.8)*	73 (98)%^#^	68 (6)	105 (22)*^#^	58 (35)%^#^
PC + T3 (*n* = 10)	7.6 (0.3)	8.3 (4.6)^#^	8 (58)%^#^	66 (4)	90 (19)*^#^	39 (27)%^#^

* *p* < 0.05 vs end of stabilization, paired samples *t*-test.

^#^ *p* < 0.05 vs NP, OneWay ANOVA.

**Figure 2:**
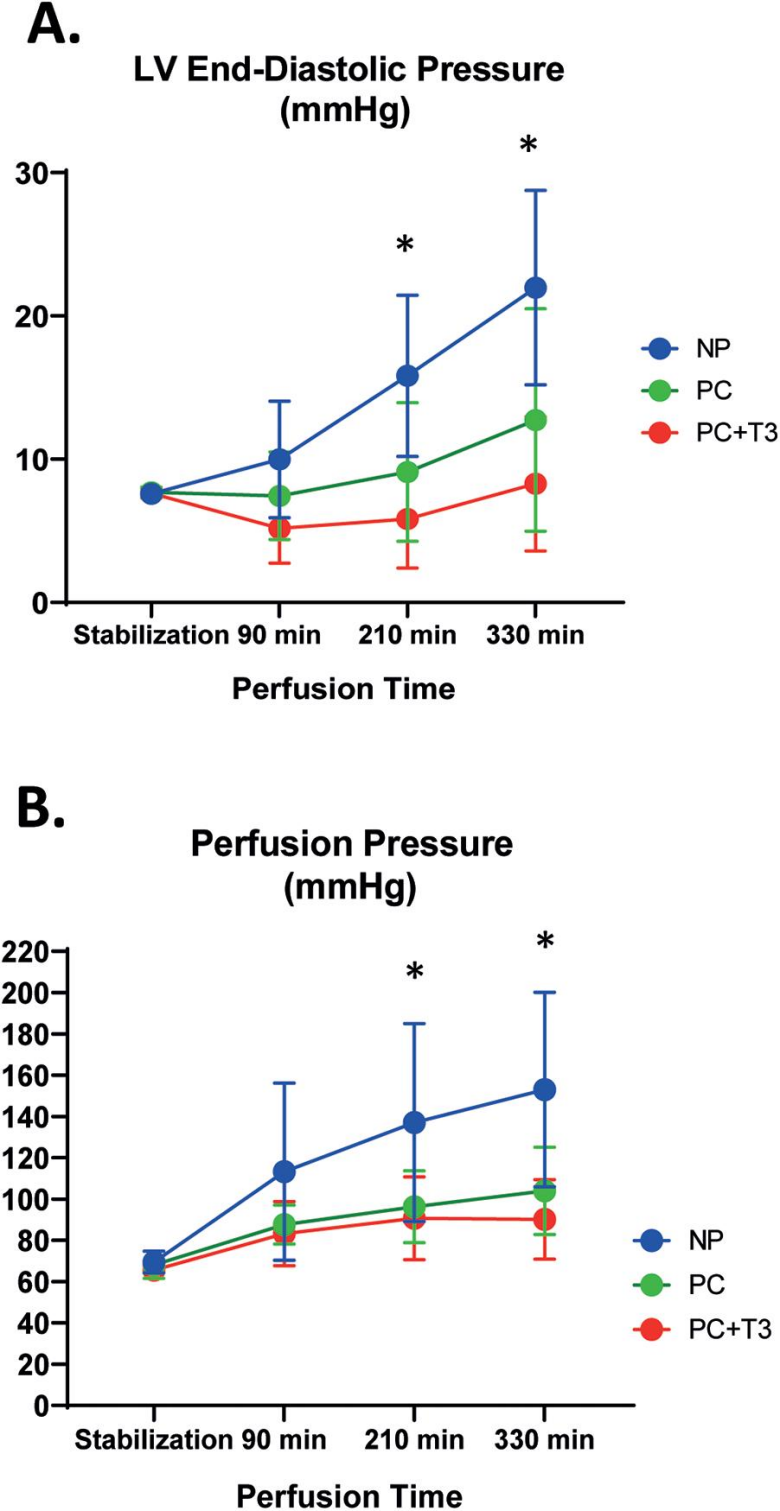
Left ventricular end-diastolic pressure (LVEDP, A), and perfusion pressure (PP, B) in hearts subjected to normothermic perfusion in langendorff apparatus for 330 min after an initial period of 30 min perfusion (NP). Another group of hearts, after stabilization for 30 min, was perfused with normal flow for 30 min followed by 5 cycles of 40 min low flow perfusion and 20 min normal flow perfusion with either vehicle (PC, *n* = 11) or 60nM T3 (PC + T3, *n* = 10) in the perfusate. Values represent means, bars stand for standard deviation. * *p* < 0.05 vs PC and PC + T3, One-way Anova with a *post hoc* analysis using Bonferroni correction.

Mixed Repeated Measures ANOVA analysis for LVEDP (dependent variable) at different time points between the groups showed that the main effect of interventions (PC and PC + T3, “between-subjects” factor) on LVEDP was statistically significant (F = 9.2, *p* = 0.001). Using Tukey *post hoc* test, LVEDP was found to be significantly lower for PC group (*p* = 0.048) and for PC + T3 (*p* = 0.0001) compared to NP. Thus, both PC and PC + T3 treatment resulted in a lower LVEDP over time. Furthermore, there was statistical significance (F = 32.5, *p* = 10^−13^) regarding the main effect of time (“within-subjects” factor) on LVEDP. There was also a statistically significant interaction effect between time and group (F = 8.9, *p* = 10^−6^). Figure 2A.

### Coronary perfusion pressure (PP)

Mean flow rate at the end of stabilization period was similar in all groups. Baseline coronary perfusion pressure was similar in all groups. At the end of perfusion, PP was significantly increased in all groups as compared to baseline (*p* < 0.05, Table 2). In NP group, PP increased 120 (63)% from baseline vs. 58 (35)% and 39 (27)% in PC and PC + T3 group, *p* = 0.02 and *p* = 0.002, respectively Table 2.

Mixed Repeated Measures ANOVA analysis for PP (dependent variable) at different time points between the groups showed that the main effect of interventions (PC and PC + T3, “between-subjects” factor) on PP was statistically significant (F = 6.6, *p* = 0.005). Using Tukey *post hoc* test, PP was found to be significantly improved for PC group (*p* = 0.035) and for PC + T3 (*p* = 0.005) compared to NP). Thus, both PC and PC + T3 treatment induced a sustained improvement of PP over time. Furthermore, there was a statistically significant effect (F = 46.9, *p* = 10^−15^) regarding the main effect of time (“within-subjects” factor) on deterioration of PP. *Α* statistically significant interaction effect between time and group-was found (F = 5.6, *p* = 0.00009). Figure 2B.

### LV weight to body weight ratio

The ratio of LV weight to body weight was found to be 2.3 (0.18) for NP group vs. 2.05 (0.15) for PC group (*p* = 0.006) and 2.1 (0.18) for PC + T3 group (*p* = 0.035 vs. NP).

## Discussion

In the present study, we used an isolated rat heart preparation perfused in a Langendorff mode as previously described in order to investigate the potential effects of interventions mimicking ischemic preconditioning on cardiac preservation under normothermic conditions (24). We established a retrograde perfusion of the heart similar to the perfusion modality used in the perfusion machines approved for human heart perfusion (2, 5) but with an acellular crystalloid buffer. In this experimental setting, during a period of 6 h perfusion, coronary perfusion pressure increased 120% from baseline indicating that microvascular dysfunction may have occurred. Furthermore, left ventricular end diastolic pressure was increased by 188%. PC treatment improved this response. PC resulted in significantly less increase in left ventricular end diastolic pressure and in coronary perfusion pressure (73% and 58% vs. 188% and 120% in NP respectively). In addition, a significant reduction in tissue edema compared to NP alone was observed. This finding is in accordance with previous reports showing that ischemic preconditioning can limit reperfusion edema (27). Increasing myocardial edema has been associated with greater microvascular obstruction and LVEDP in the reperfused human myocardium (28). Based on this evidence, the PC effect on coronary pressure and LVEDP could, at least in part, be attributed to less edema which was observed in PC perfused hearts. Ischemic preconditioning has been shown to act via vasodilatory mediators such as adenosine and prostacyclin which may be involved in the observed PC-induced cardioprotection (29). In fact, in a retrograde rat heart perfusion model with acellular buffer, adenosine was shown to decrease edema formation, inflammation and vascular resistance (26). Interestingly, the combination of adenosine with low pressure acellular perfusion was found to be an optimal heart preservation modality (26).

The present study also showed that systolic function declined after 6 h perfusion and this response was not improved by PC. The mechanisms of this phenomenon are not fully understood. One plausible explanation is that ischemic preconditioning causes stunning and reversible decline of systolic function, whereas at the same time protects the heart from injury (30, 31). This issue merits further investigation.

A crystalloid based perfusate was used in this study rather than conventional blood-based perfusates currently used in clinical practice. It is now realized that crystalloid or blood based solutions can be improved by changes in the metabolic substrates in the perfusate (7, 8). Glucose seems to be an ineffective metabolic substrate for the reperfused myocardium due to the uncoupling of glycolysis to glucose oxidation even if insulin is included in the perfusate (7, 8). Replacement of glucose by pyruvate improved myocardial metabolism and optimized cardiac perfusion (7, 8). Along this line, enhancing the coupling of glycolysis to glucose oxidation may be an alternative approach. Notably, thyroid hormone and ischemic preconditioning are shown to enhance glucose homeostasis upon reperfusion (9, 32). On the basis of this evidence, we chose glucose as the only metabolic substrate in the perfusate as in our previous study (24). This crystalloid solution enriched with high dose triiodothyronine was previously shown to protect the isolated perfused rat heart against myocardial and vascular dysfunction (24).

Taken together, these data provide evidence that interventions mimicking ischemic preconditioning may be an effective means of cardioprotection for hearts exposed to long periods of normothermic perfusion. However, ischemic preconditioning response may vary depending on the metabolic substrate of the perfusate or the status of the perfused heart. The presence of pyruvate can increase the threshold for preconditioning and low glucose concentration can attenuate its effect (32, 33).

The response of the heart to ischemic preconditioning appears to be dependent on the thyroid status and low thyroid hormone can attenuate its effect (34, 35). This may be of clinical relevance since severe damaged hearts are depleted from thyroid hormone (10) and a combination of PC with T3 may be a more appropriate treatment. Thus, in the present study, we also investigated the PC effect when T3 was added in the perfusate. Our data showed that the combination of PC and T3 can also confer protection as PC alone. Furthermore, PC in combination with T3 did not improve the systolic function but had a favourable effect on the diastolic function. T3 and ischemic preconditioning can confer cardioprotection via their effect on metabolism and vasodilation but also through their action on hypoxia-induced intracellular signalling pathways (12, 22, 32, 36, 37). Oxidative stress occurs upon oxygen restoration in the postischemic heart resulting in activation of pro-apoptotic pathways which mediate endothelium and cardiomyocyte dysfunction and damage (15, 16). Thus, the proapoptotic p38 MAPK, which regulates SERCA (Sarcoplasmic Reticulum Calcium ATPase) function, is activated upon reperfusion resulting in changes in calcium homeostasis and cardiac diastolic dysfunction (14). Furthermore, activation of p38 MAPK can induce endothelial barrier dysfunction and vascular leakage leading to microvascular obstruction due to mechanical compression (15). In line with this, p38 MAPK inhibitors applied during organ procurement and storage can protect the graft against ischemia-reperfusion injury (36). In this context, we have previously shown that triiodothyronine suppressed the activation of proapoptotic signalling pathways in hearts exposed to normothermic perfusion and this response was associated with enhanced heart preservation (24). Furthermore, T3 suppressed the proapoptotic caspase-3 and prevented cold-warm reperfusion injury in lung epithelial cells (38).

### Limitations of the study

Some limitations in the present study should be taken into account. In this study, an acellular crystalloid based perfusate was used rather than a blood-based perfusate currently used in clinical practice. Crystalloid buffers have traditionally been used in Langendorff perfusion to study rodent heart physiology and pharmacology, but their use is limited in human heart perfusion due to low oxygen solubility particularly with increasing temperature in the perfusate (11). Although perfusion with blood-based solutions is considered superior to crystalloid based perfusion, an increased risk of microvascular obstruction may occur in the setting of ischemia -reperfusion due to erythrocyte aggregation (11, 39, 40). In accordance with the above, in a Langendorff rat heart normothermic perfusion for 4 h, the use of packed red blood cells induced a pro-inflammatory response associated with a greater cardiac damage compared to acellular perfusion (26). Blood perfusion imposes several technical challenges and make the perfusion system more complex. Thus, the interest for crystalloid based normothermic perfusion modalities has recently been revived highlighting the importance of the present findings. It should be noted that from a translational perspective, a normothermic acellular perfusate-based ex vivo system does not yet exist for heart transplantation. The present study has been designed to provide evidence for translational studies in large animals and humans. In this context, molecular mechanisms have not been investigated. However, the mechanisms of actions of ischemic preconditioning and T3 on reperfused myocardium have extensively been studied in previous investigations (22, 41). Finally, our experimental model used hearts from animals after anaesthesia and not after brain death. Ischemic preconditioning effect may be limited in hearts harvested from brain death. However, previous investigations have shown that the ischemic preconditioning response was preserved in hearts obtained after brain death and transplanted to the recipient animals (42).

In conclusion, intermittent low flow mimicking ischemic preconditioning appears to optimize crystalloid normothermic rat heart perfusion by limiting tissue edema and diastolic and vascular dysfunction. This study may be a paradigm for further investigations to explore the potential of simple crystalloid solutions in combination with means of cardioprotection to optimize normothermic perfusion.

## Data Availability

The raw data supporting the conclusions of this article will be made available by the authors, without undue reservation.
